# Sex-specific and polygenic effects underlying resting heart rate and associated risk of cardiovascular disease

**DOI:** 10.1093/eurjpc/zwae092

**Published:** 2024-03-04

**Authors:** Ada N Nordeidet, Marie Klevjer, Karsten Øvretveit, Erik Madssen, Ulrik Wisløff, Ben M Brumpton, Anja Bye

**Affiliations:** Cardiac Exercise Research Group (CERG), Department of Circulation and Medical Imaging, Faculty of Medicine and Health Sciences, Norwegian University of Science and Technology (NTNU), Prinsesse Kristinas gate 3, 7030 Trondheim, Norway; Cardiac Exercise Research Group (CERG), Department of Circulation and Medical Imaging, Faculty of Medicine and Health Sciences, Norwegian University of Science and Technology (NTNU), Prinsesse Kristinas gate 3, 7030 Trondheim, Norway; Department of Cardiology, St. Olavs Hospital, Trondheim University Hospital, Trondheim, Norway; K.G. Jebsen Center for Genetic Epidemiology, Department of Public Health and Nursing, Faculty of Medicine and Health Sciences, Norwegian University of Science and Technology (NTNU), Trondheim, Norway; Department of Cardiology, St. Olavs Hospital, Trondheim University Hospital, Trondheim, Norway; Cardiac Exercise Research Group (CERG), Department of Circulation and Medical Imaging, Faculty of Medicine and Health Sciences, Norwegian University of Science and Technology (NTNU), Prinsesse Kristinas gate 3, 7030 Trondheim, Norway; Centre for Research on Exercise, Physical Activity and Health, School of Human Movement and Nutrition Sciences, University of Queensland, St. Lucia, Brisbane, Queensland, Australia; K.G. Jebsen Center for Genetic Epidemiology, Department of Public Health and Nursing, Faculty of Medicine and Health Sciences, Norwegian University of Science and Technology (NTNU), Trondheim, Norway; Cardiac Exercise Research Group (CERG), Department of Circulation and Medical Imaging, Faculty of Medicine and Health Sciences, Norwegian University of Science and Technology (NTNU), Prinsesse Kristinas gate 3, 7030 Trondheim, Norway; Department of Cardiology, St. Olavs Hospital, Trondheim University Hospital, Trondheim, Norway

**Keywords:** Heart rate/genetics, Genome-wide association study/methods, Cardiovascular diseases/genetics, Risk factors, Polymorphisms, single nucleotide, Atrial fibrillation, Cardiomyopathy, dilated, Hypertension

## Abstract

**Aims:**

Resting heart rate (RHR) is associated with cardiovascular disease (CVD) and mortality. This study aimed to identify genetic loci associated with RHR, develop a genome-wide polygenic risk score (PRS) for RHR, and assess associations between the RHR PRS and CVD outcomes, to better understand the biological mechanisms linking RHR to disease. Sex-specific analyses were conducted to potentially elucidate different pathways between the sexes.

**Methods and results:**

We performed a genome-wide meta-analysis of RHR (*n* = 550 467) using two independent study populations, The Trøndelag Health Study (HUNT) and the UK Biobank (UKB), comprising 69 155 and 481 312 participants, respectively. We also developed a genome-wide PRS for RHR using UKB and tested for association between the PRS and 13 disease outcomes in HUNT. We identified 403, 253, and 167 independent single nucleotide polymorphisms (SNPs) significantly associated with RHR in the total population, women, and men, respectively. The sex-specified analyses indicated differences in the genetic contribution to RHR and revealed loci significantly associated with RHR in only one of the sexes. The SNPs were mapped to genes enriched in heart tissue and cardiac conduction pathways, as well as disease-pathways, including dilated cardiomyopathy. The PRS for RHR was associated with increased risk of hypertension and dilated cardiomyopathy, and decreased risk of atrial fibrillation.

**Conclusion:**

Our findings provide insight into the pleiotropic effects of the RHR variants, contributing towards an improved understanding of mechanisms linking RHR and disease. In addition, the sex-specific results might contribute to a more refined understanding of RHR as a risk factor for the different diseases.

## Graphical Abstract

**Graphical Abstract:**
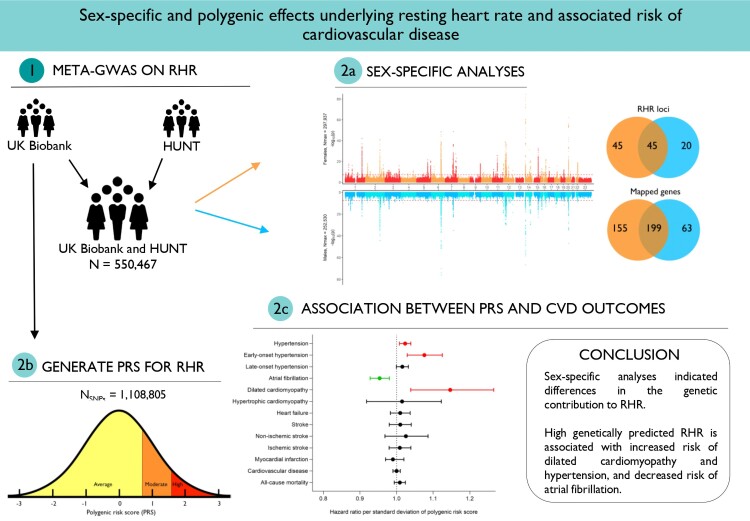

## Introduction

Cardiovascular disease (CVD) is the leading cause of mortality worldwide and accounts for twice as many deaths in Europe than all cancers combined.^1^ Hence, advancing our understanding of the underlying mechanisms of CVD has great potential in decreasing the global CVD burden. Resting heart rate (RHR) is an independent risk factor for CVD, and this relationship has been demonstrated in multiple subgroups including patients with hypertension, coronary artery disease including myocardial infarction (MI), heart failure (HF), atrial fibrillation (AF), and stroke, but also in healthy individuals.^2–5^ In addition, RHR is a predictor of overall mortality.^6,7^ In general, a lower RHR implies a more efficient heart function and a better cardiovascular fitness.

Resting heart rate is a complex trait regulated by interactions between several biological systems, including the sinus node, autonomous nervous system, central cortex, baroreceptors, and cardiac regulatory mechanics.^8,9^ The genetic contribution to RHR is estimated to be ∼22% in family studies and up to 65% in twin studies.^10–12^ From the technological development in genotyping, there has been an almost exponential increase in genome-wide association studies (GWASs). Until recently, the largest GWAS of RHR was conducted on ∼428 000 participants from the UK Biobank (UKB), resulting in the identification of 437 independent loci associated with RHR.^13^ However, van de Vegte *et al*.^14^ newly published a genome-wide meta-analysis of RHR including 100 studies and ∼835 000 participants, identifying 493 independent loci including 68 novel genetic variants.

A way of applying the large amount of data available from GWAS is to calculate a polygenic risk score (PRS), which is an estimate of an individual’s genetic susceptibility to a specific disease or trait. A PRS is typically calculated as the number of risk alleles carried, weighted by the effect of each genetic marker, summarized to a single score. Recently, a PRS for RHR was derived from a small study population (*n* = 1280), consisting of 80 genetic markers that explained 1.46% of the variance in RHR.^15^ Novel methods incorporating a larger number of genetic effect estimates may improve the predictive power of highly polygenic traits such as RHR.^16^

In this current study, we aimed to investigate the genetic architecture of RHR to gain insight into underlying mechanisms and how it relates to different CVDs. To achieve this, we conducted a genome-wide meta-analysis of RHR, followed by functional interpretation and annotation of the results. Further on, we constructed a genome-wide PRS for RHR and tested for association with multiple CVD outcomes to assess if genetic susceptibility to high or low RHR is associated with disease risk. We performed sex-specific analyses to shed light on the possible sex-specific genetic architecture of RHR and its relation to disease.

## Methods

### Study participants from The Trøndelag Health Study (HUNT)

HUNT is one of the largest health studies ever performed. The HUNT study has collected questionnaire data, clinical measurements, and biological samples from ∼229 000 participants through four surveys HUNT1 (1984–86), HUNT2 (1995–97), HUNT3 (2006–08), and HUNT4 (2017–19).^17,18^ DNA extracted from blood samples has been subject to large-scale genotyping and imputation resulting in more than 24.9 million well-imputed single nucleotide polymorphisms (SNPs). Genotyping, imputation, and sample and quality control were performed by standard protocols and is described in detail elsewhere.^19^ In the present study, we included 69 155 participants from HUNT2 and HUNT3 with genotype data and heart rate measures. For the sex-specific analyses, there were 36 595 women and 32 560 men. In each HUNT survey, heart rate was measured as beats per minute (b.p.m.) at three time points, and the lowest heart rate measure was defined as RHR. If a participant underwent heart rate measures in both HUNT2 and HUNT3, the measures from HUNT2 were used. Written informed consent was obtained from all participants included in the study.

### Study participants from the UK Biobank (UKB)

The UKB consists of more than 500 000 participants aged 40–69 enrolled between 2006 and 2010. All 500 000 participants have been genotyped and imputed providing information on more than 90 million SNPs. The study and participants have been described in detail elsewhere.^20^ Resting heart rate was assessed by an automated reading during blood pressure measurement (ID fields 95 and 102), and by the pulse rate obtained from arterial stiffness measurement (ID field 4194). If several measurements were available for one participant, the RHR was defined as the lowest measured heart rate using ID fields 102, 4194, and 95. All participants with genotype data and pulse measurements available were included resulting in 481 312 participants, including 261 342 women and 219 970 men for the sex-specific analyses. Written informed consent was obtained from all participants included in the study.

### Statistical analyses genome-wide association study

The GWAS was performed using BOLT-LMM (v.2.3.4), a linear mixed model that accounts for population stratification and relatedness and hence increases power as related samples do not need to be excluded.^21^ The analyses were adjusted for age, sex, principal components (PCs) 1–10, and genotyping batch. In addition, sex-specific analyses were conducted, adjusted for age, PC1–10, and genotyping batch.

### Meta-analysis

The GWAS results from HUNT and UKB were meta-analysed using METAL, an efficient method of analysing genome-wide association summary statistics, which allows for increased statistical power compared to single GWAS.^22^ The increased statistical power provides an opportunity to detect significant variants with modest effect sizes. METAL was used to conduct meta-analysis for the total population, as well as separate analyses for women and men. The meta-analyses included SNPs with minor allele frequency > 0.001. Genomic control correction of any analyses with an inflation factor λ > 1 was performed. The genetic loci that reached a *P*-value of <5 × 10^−8^ are reported as significant findings.

### Identification of risk loci and lead SNPs

To identify RHR risk loci and lead SNPs from the genome-wide meta-analyses, we used FUMA (v.1.5.4), a web-based platform combining information from multiple biological resources.^23^ The *R*^2^ threshold was set to 0.6 to define independent significant SNPs, which were used to determine the borders of the genomic risk locus. Clumping of the independent significant SNPs was performed to identify lead SNPs independent from each other (*R*^2^ ≤ 0.2). For the identification of RHR risk loci, the maximum distance for linkage disequilibrium (LD) blocks to merge was set to 250 kb.

### Gene mapping and functional annotation of the genome-wide association study results

The results from the genome-wide meta-analyses were further explored using functional annotation and gene mapping by SNP2GENE and GENE2FUNC integrated in FUMA.^23^ Positional gene mapping was performed based on ANNOVAR annotations, and tissue specificity of mapped genes was assessed through an enrichment test for differentially expressed genes (DEGs) as implemented in GENE2FUNC. The GENE2FUNC also test the mapped genes for enrichment in pre-defined gene sets from MsigDB, KEGG, WikiPathways, and the GWAS Catalog. Significant enrichment at Bonferroni corrected *P*-value of ≤0.05 was reported as significant findings.

### Polygenic risk scores for resting heart rate

We used the results from our UKB GWAS to develop a genome-wide PRS for RHR. The Bayesian approach PRS-CS-auto was applied to learn the optimal global shrinkage parameter directly from the base data, which has been shown to work well with large sample sizes.^24^ A PRS containing 1 108 805 genetic variants was derived and applied downstream in an independent dataset consisting of all four HUNT surveys (*n* = 86 687). The PRS was also applied in women (*n* = 45 970) and men (*n* = 40 717) separately, to test for association with disease outcomes. For comparative analyses, we also constructed a weighted PRS using the 80 variants previously reported by Xie *et al*.^15^ (PRS_80_) and applied it in the same sample.

### Disease outcomes in HUNT

Disease outcomes were available for all participants in all four HUNT surveys through health registry data spanning from January 1999 throughout March 2020. The 10th revision of the International Statistical Classification of Diseases and Related Health Problems (ICD-10) codes derived from the Nord-Trøndelag Hospital Trust were used to construct disease outcomes relevant for RHR including hypertension, early-onset hypertension (<55 years old), late-onset hypertension (>55 years old), AF, dilated cardiomyopathy (DCM), hypertrophic cardiomyopathy, HF, stroke, non-ischaemic stroke, ischaemic stroke, MI, CVD, and all-cause mortality. ICD-10 codes used for the classification and disease prevalence are found in Supplementary material online, *Tables S1* and *S2*.

### Statistical analysis for polygenic risk score

Statistical analyses were performed using R (v.4.2.3). Pearson’s correlation coefficient was used to estimate the correlation between the two PRSs and RHR measurements in HUNT. Cox proportional hazard models were used to assess the impact of PRS on disease risk. The models were adjusted for sex and first 10 PCs, with age as the time scale.

## Results

The mean RHR in HUNT was 69.9 b.p.m. (SD = 12.4), while the mean RHR in UKB was 67.1 b.p.m. (SD = 11.1). The distribution of RHR can be found in Supplementary material online, *Figure S1*. The number of participants, genome-wide significant SNPs, RHR risk loci and lead SNPs for each of the population in the genome-wide meta-analyses is presented in *Table 1* (details in Supplementary material online, *Tables S5*–*S10*). We identified one novel RHR locus in women on chromosome 2, with lead SNP rs4666343 (*P* = 3.9 × 10^−8^, effect size = −0.1954), not previously reported to be associated with RHR (>1 Mb away from previously reported loci). All other identified RHR loci are consistent with previous findings.

**Table 1:** A presentation of the number of participants, number of genome-wide significant SNPs, and number of lead SNPs for each of the populations in the genome-wide meta-analysis

Population	Number of participants	Number of genome-wide significant SNPs	Number of RHR risk loci	Number of lead SNPs
Total	550 467	13 890	141	403
Women	297 937	10 231	90	253
Men	252 530	6016	60	167

### Exploration of the biological role of resting heart rate

The RHR SNPs were mapped to 575, 355, and 263 genome-wide significant protein-coded genes for the total population, women, and men, respectively (see Supplementary material online, *Tables S11*–*S13*). The enrichment test for DEGs implemented in GENE2FUNC was used to assess tissue specificity of the mapped genes. The enrichment test uses 54 specific tissue types to evaluate whether the mapped genes are overrepresented in DEGs. In the total population, there were significantly up-regulated DEGs in the atrial appendage of the heart, left ventricle, and skeletal muscle. In women, there were significant up-regulated DEGs in the atrial appendage of the heart and left ventricle. In men, there were significant up-regulated DEGs of the atrial appendage of the heart.

Using GENE2FUNC, hypergeometric tests were performed to test if the mapped genes were overrepresented in any pre-defined gene sets obtained from publicly available datasets. Genes mapped in the total population, women and men were enriched in some of the same gene sets. Significant findings included biological processes related to the cardiac conduction system including cardiac contraction, regulation of heart rate and blood circulation, as well as development of muscle and heart tissue (see Supplementary material online, *Tables S14*–*S16*). Mapped genes were also overrepresented in pathways of cancer, heart development defects, cardiomyopathy including DCM, metabolic regulation, G-protein signalling, and calcium signalling in cardiac cells. In women and men, the mapped genes were enriched in pathways of vasodilation, platelet formation, and neurotransmission.

### Sex differences

**Figure 1:**
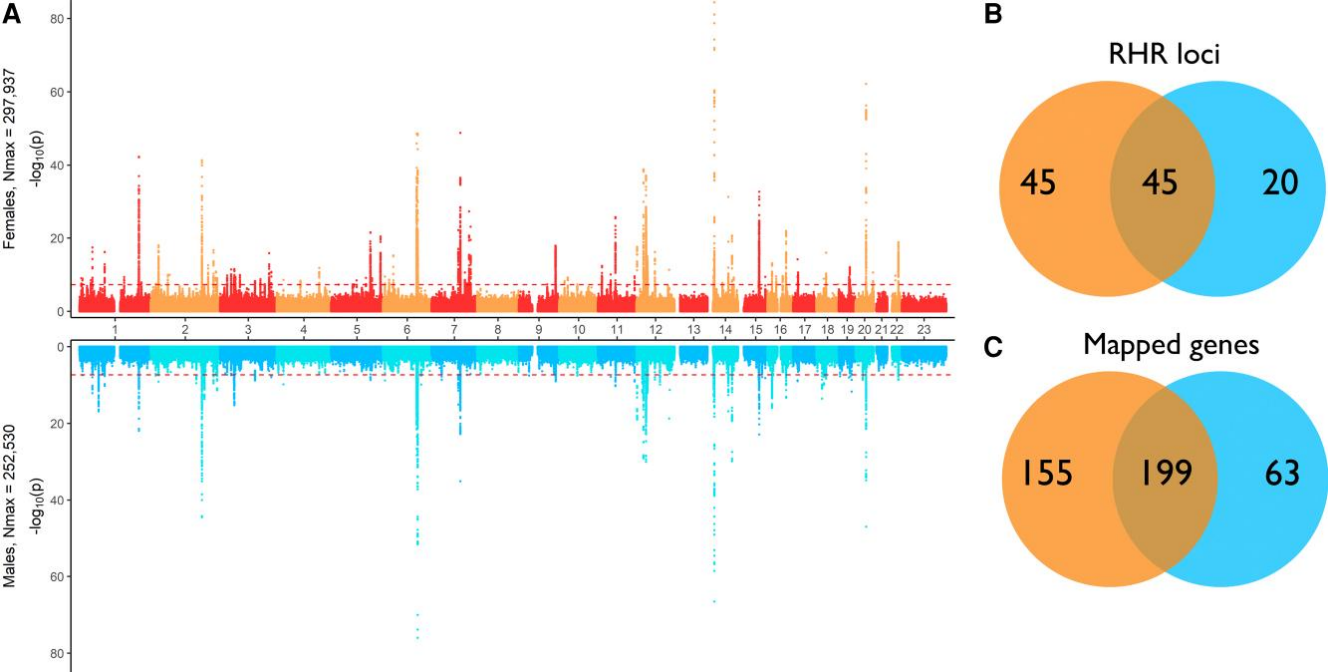
To the left (*A*) is a Miami plot of the genetic variants associated with RHR for women at top, and men at bottom. The *x*-axis displays the genetic position, and the *y*-axis shows the negative logarithm of the *P*-value. The dotted line is the genome-wide significant threshold of 5 × 10^−8^. To the right is the number of RHR loci (*B*) and mapped genes (*C*) for women and men, including number of overlapping loci and genes.

There were differences in the genetic contribution to RHR between women and men, and this is illustrated through a Miami plot in *Figure 1*. In general, the genetic contribution to RHR seems stronger in women than in men, as there were more significant SNPs, more genomic risk loci and the SNPs were mapped to more protein-coding genes in women compared to men. Of the 90 risk loci in women and 66 risk loci in men, 45 were overlapping. The loci associated with RHR only in one sex are listed in Supplementary material online, *Tables S3* and *S4*.

For ∼70% of the RHR loci identified in both women and men, the lead SNP in women reached a higher significance level compared to the lead SNP in men. On chromosome 9, one risk locus in women is overlapping with a risk locus in men, and *MAPKAP1* is mapped to the regions. The lead SNP in women has a stronger association with RHR (*P* = 1.3 × 10^−18^, effect size = −0.2917) compared to the lead SNP in the same region in men (*P* = 1.5 × 10^−8^, effect size = −0.2253). Note that the lead SNP in each respective population is not the same SNP. The same applies to a region on chromosome 11 where the *P*-value for the lead SNP in women was 2.0 × 10^−26^ (effect size = 0.3729) and the *P*-value for the lead SNP in men was *P* = 5.6 × 10^−11^ (effect size = −0.276). Genes mapped to both risk loci were *MYRF*, *TMEM258*, *FEN1*, *FADS2*, and *FADS1*.

A risk locus at the end of chromosome 5 was identified in women, with the lead SNP 5:172653978_C/CT (*P* = 3.855 × 10^−21^, effect size = 0.4509) and there was no corresponding signal in men. Genes mapped to this locus were *CREBRF*, *NKX2–5*, and *BNIP1*, and the locus included a missense variant (rs2277923) in *NKX2–5*. Two regions on chromosome 7 were also only identified in women. The lead SNPs were 7:130965408_AT/A (*P* = 5.7 × 10^−28^, effect size = 0.3737) and 7:136595547_A/AGT (8.5 × 10^−24^, effect size = 0.4573), respectively, and the genes *CHRM2*, *MKLN1*, and *PODXL* were mapped to the regions.

Resting heart rate loci only identified in men included a risk locus on chromosome 1, with lead SNP rs10789207 (*P* = 1.5 × 10^−17^, effect size = −0.3807), and genes mapped to the region were *LEPR*, *PDE4B*, *SGIP1*, *AL139147.1*, *TCTEX1D1*, *INSL5*, *WDR78*, and *MIER1*. Another region specific to men was a risk locus on chromosome 19, with lead SNP rs1065853 (*P* = 2.1 × 10^−12^, effect size = −0.5134), and mapped genes *APOE*, *TOMM40*, and *APOC1*. One of the significant SNPs in the locus, rs7412, is a missense variant in *APOE*. A third locus with lead SNP rs13384908 (*P* = 2.324e^−14^, effect size = −0.3506) on chromosome 2 included *CALCR* and *TFPI*.

### Polygenic risk score

**Figure 2:**
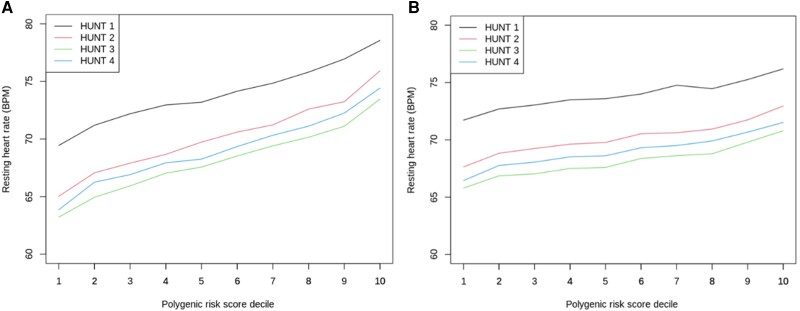
Genotype–phenotype associations for the genome-wide polygenic risk score to the left (*A*) and the 80-variant polygenic risk score to the right (*B*). HUNT1 (*n* = 45 215), HUNT2 (*n* = 57 872), HUNT3 (*n* = 43 182), HUNT4 (*n* = 51 177).

**Figure 3:**
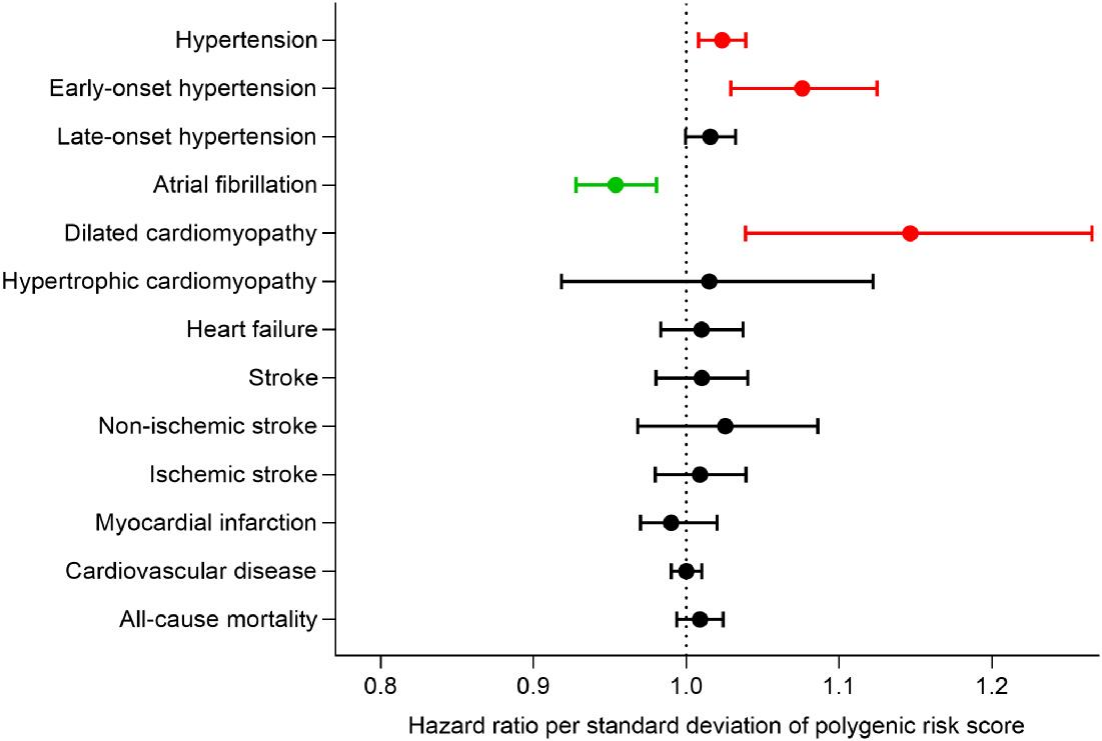
Hazard ratios for cardiovascular outcomes.

Both PRSs were associated with RHR in all four HUNT surveys (*Table 2*; *Figure 2*). The genome-wide PRS had an over twice as strong phenotypic correlation compared to the PRS_80_. The genome-wide PRS analyses showed that a genetic susceptibility to high RHR was associated with an increased risk for hypertension, early-onset hypertension, and DCM, and a decreased risk of AF (*Figure 3*).

**Table 2:** The phenotypic correlation between the polygenic risk score and resting heart rate in the different HUNT surveys

	PRS		PRS_80_		
	*r*	*P*	*r*	*P*	Sample size
HUNT 1	0.220	<2.2e^−16^	0.104	<2.2e^−16^	45 215
HUNT 2	0.247	<2.2e^−16^	0.116	<2.2e^−16^	57 872
HUNT 3	0.262	<2.2e^−16^	0.123	<2.2e^−16^	43 182
HUNT 4	0.251	<2.2e^−16^	0.119	<2.2e^−16^	51 177

HUNT, The Trøndelag Health Study; PRS_80_, polygenic risk score consisting of 80 previously identified variants; PRS, genome-wide polygenic risk score consisting of 1 108 805 variants derived from our UKB GWAS results; *r*, Pearson’s correlation coefficient; *P*, *P*-value.

**Figure 4:**
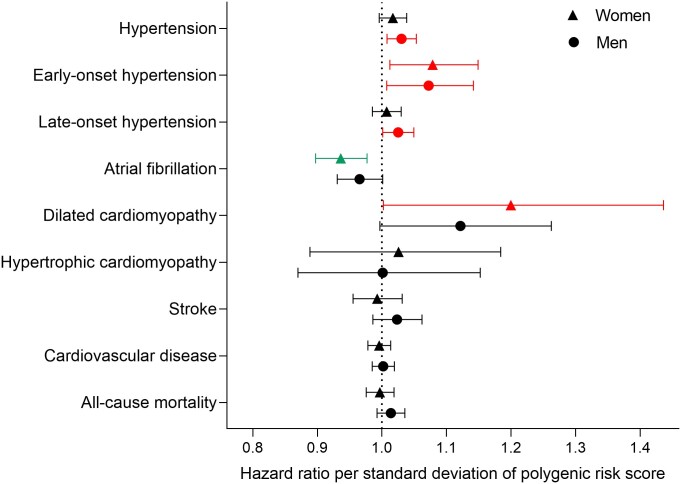
Hazard ratios for cardiovascular outcomes in women and men separately.

The PRS was also tested in women and men, separately, and revealed sex-specific associations with CVD outcomes (*Figure 4*). In men, a high RHR PRS was associated with higher risk of hypertension, early-onset hypertension, and late-onset hypertension. In women, a high RHR PRS was associated with higher risk of early-onset hypertension and DCM, and lower risk of AF.

## Discussion

We have conducted a large-scale genome-wide meta-analysis, identifying 403, 253, and 167 lead SNPs associated with RHR in the total population, women, and men, respectively. We identified several loci significantly associated with RHR only in one sex, which might imply sex-specific genetic architecture. Additionally, we have created a genome-wide PRS for RHR that improves upon previous efforts. The RHR PRS created in this present study was associated with an increased risk of hypertension, including early-onset hypertension, and DCM as well as a decreased risk of AF. This indicates shared genetics between RHR and these disease outcomes. In addition, when testing the PRS in women and men separately, we found differences in associations to disease risk. To our knowledge, this is the first study focusing on potential sex-specific genetic markers for RHR. Our findings advance the current understanding of the genetic architecture of RHR and its association with CVD.

A high RHR PRS was associated with increased risk of hypertension and especially early-onset hypertension. This is of particular interest, as early-onset hypertension is associated with greater risk of CVD mortality compared to hypertension later in life.^25–27^ The HARVEST study found that both RHR and changes in RHR during 6 months follow-up predicted sustained hypertension for patients with early-onset hypertension.^28^ In general, high heart rate is considered a predictor of hypertension progression and one study found that participants in the highest RHR quartile had a 16% higher risk of hypertension.^29^ Our finding is consistent with a previous GWAS reporting an association between genetically determined RHR and hypertension.^30^ One possible explanation of the link between RHR and hypertension is the sympathetic part of the autonomic nervous system. Elevated RHR may indicate sympathetic overactivation, which can contribute to increased blood pressure through different pathways, including an increase in vasoconstriction of resistance blood vessels, thus resulting in an increased blood pressure.^31,32^ It can also lead to sustained stress to the arterial wall, resulting in arterial stiffening^33^ that may predispose to hypertension, as well as other CVDs.

In general, elevated RHR is associated with greater disease risk, but in the case of AF, the relationship is still unclear. Observational studies have reported a U-shaped association between RHR and AF,^5^ while linear Mendelian Randomization (MR) studies have reported an inverse association.^34–36^ Two recent non-linear MR studies, looking at non-linear effects, have confirmed this linear inverse association in the MR setting, at least for RHR values up to 90–98 b.p.m.^14,37^ The PRS analyses in the present study showed a decreasing risk of AF with genetic susceptibility to higher RHR, supportive of the MR results. The underlying mechanisms causing the increased risk of AF for low RHR values are still unclear. One potential mechanism regulating this association is the autonomic tone, which is suggested to be central in the pathophysiology of HF, hypertension, and arrhythmias.^36,38^ Vagal stimulation in the atrial myocardium is arrhythmogenic and could lead to AF. Interestingly, increased physical activity is associated with a lower RHR, and it is shown that high exercise volumes are associated with an increased risk of AF, and there is a high prevalence of AF in endurance athletes, especially in men.^39–41^ High exercise volumes over many years might induce cardiac adaptations including cardiac remodelling, atrial enlargement, and left ventricular hypertrophy that could lead to an increased risk of AF.^42–44^ Other mechanisms that could explain the genetic link between RHR and AF involve atrial dilation, fibrosis, increased atrial ectopic activity, greater parasympathetic activation, and blunted sympathetic tone.^39,44^

A high genetically predicted RHR was also significantly associated with an increased risk of DCM, consistent with the recent results from the MR analyses of van de Vegte *et al*.^14^ Dilated cardiomyopathy is a heart muscle disease with structural and functional abnormalities in the myocardium and is characterized by ventricular dilation.^45^ A possible mechanism explaining the association between genetically high RHR and DCM could involve tachycardia-mediated cardiomyopathy, a reversible form of DCM that might be caused by a long-standing elevated RHR.^46^ The genetic architecture of DCM is complex, but some candidate genes have been established. A systematic study found 19 genes with strong evidence of a role in DCM^47^ and interestingly, seven of these genes (*DES*, *MYH7*, *PLN*, *RBM20*, *SCN5*, *TTN*, and *DSP*) were among the mapped RHR genes in this study, indicating shared genetics between RHR and DCM. The RHR PRS was not significantly associated with hypertrophic cardiomyopathy, and a potential explanation could be low statistical power due to the low prevalence in HUNT (*n* = 382). However, we detected an association to DCM despite few cases (*n* = 394), making it more likely that these cardiomyopathy subgroups display different genetic architecture since they are characterized by different structural and functional changes in the myocardium.^48^

Further on, the RHR PRS was tested against CVD outcomes separately in women and men, and interestingly there were differences in associations with disease. It appears that the RHR variants constituting the PRS for the total population may have different effects on disease risk in women and men. Even though there are more women than men in HUNT [45 970 (53.03%) vs. 40 717 (46.97%)], it is likely that other factors than the sample size explain the differences. The RHR PRS was associated with early-onset hypertension in women, but it was associated with all hypertension outcomes in men, including late-onset hypertension despite the lower sample size and lower number of cases in men (*n* = 6978 vs. *n* = 7820 in women). Further, the increased risk of DCM in the total population was attenuated and not statistically significant in men (*n* = 279), but more significant in women even though there were fewer cases (*n* = 118), and thus less statistical power. The association with AF in the total population was present in women, but the association was not significant in men. Even though the association with AF was not statistically significant in men, the hazard ratio and confidence interval do indicate that high PRS is associated with lower risk of AF, thus underlining why we must be cautious about drawing final conclusions on sex differences from these results. In general, conducting sex-specific analyses reduce the sample size by half, leading to less statistical power to detect significant findings. Except for late-onset hypertension in women, all significant associations in the total population that were not significant in the sex-specific analyses were marginally non-significant, suggesting a weak association also in each of the sexes.

The results from the sex-specific genome-wide meta-analyses identified loci that were significantly associated with RHR only in one sex, which might point towards sex-specific biology. We also found more significant RHR variants in women compared to men that might imply a stronger genetic contribution to RHR in women than in men. It is likely that the genetic contribution to RHR might be different between the sexes as the female and male heart differs in mass, size, function, hormonal features and structure.^49^ In general, women have a higher RHR and a longer QT interval (i.e. time from contraction to relaxation) than men.^49,50^ This is due to the role of testosterone in ventricular repolarization, and it results in a generally higher risk of drug-induced arrhythmias in women.^49,51^ The role of sex hormones in the progression of CVD has recently received attention, but their effects are not well-understood.^52^ Genotype by sex interaction is thought to account for some of the differences seen between women and men in complex traits and the risk of disease, and it might apply to RHR. Bernabeu *et al*.^53^ found significant genetic heritability differences between women and men in ∼50% of the binary and 7% of the quantitative trait they examined in the UKB. Our findings emphasize the importance of considering sex when assessing the genetics of RHR and how it relates to CVD.

A RHR locus on chromosome 5 identified in women, and not in men, included the gene *NKX2–5* and the *NKX2–5* missense variant rs2277923. NKX2–5 is a transcription factor crucial for regulation of cardiac development,^54^ and genetic variants in *NKX2–5*, including rs2277923, are found to be associated with congenital heart disease.^55^ Notably, one study assessing sex-specific SNPs in patients with a bicuspid aortic valve found variants in *NKX2–5* specific to women.^56^ Together with that finding, our results might indicate that *NKX2–5* variants have an effect on RHR and possibly congenital heart disease in women. The RHR locus on chromosome 7 only identified in women included the mapped gene *CHRM2* that encodes M2 muscarinic acetylcholine receptor (M2R). M2R is the most important cardiac receptor of the parasympathetic nervous system and influence heart rate and contractility.^57^ Antibodies reacting with the M2R were found to contribute to the pathogenesis of several CVDs, including DCM and arrhythmic disorders such as AF,^58–60^ which is interesting as the RHR PRS was associated with DCM and AF in women. *CHRM2* was exclusively mapped in women suggesting its potential significance for RHR and possibly CVD among women.

One of the genomic loci identified in men and not in women included the calcitonin receptor-like (*CALCR*) gene. *CALCR* encodes for a G-protein coupled receptor that links to a receptor activity modifying protein to compose calcitonin gene-related peptide (CGRP). CGRP is a potent vasodilator and is involved in the regulation of blood pressure.^61^ We can speculate that genetic variation in *CALCR* contributes to the association between RHR and hypertension found in this study. It appears to be of significance for men, which is interesting as the PRS was associated with all three hypertension outcomes in men, but only with early-onset hypertension in women. Notably, studies have suggested that oestrogen might influence the CGRP levels,^62,63^ further indicating a need for considering sex differences when studying *CALCR* in CVD. Another genomic locus only identified in men included the gene *APOE* and the missense variant rs7412. APOE (Apolipoprotein E) is involved in the fat metabolism and is implicated in regulation of triglycerides, cholesterol, and low-density lipoprotein cholesterol, which are well established CVD risk factors.^64,65^ A previous study reported an association between genetic variants in *APOE* and the risk of MI.^65^*APOE* is a well-known risk gene in Alzheimer’s disease (AD).^64,65^ Notably, a previous study reported that sex modifies the *APOE*-related risk of disease, as they found that *APOE* may play a more prominent role in the AD development in women compared to men.^66^ Our findings might indicate that the genetic impact of *APOE* on RHR, and perhaps disease, is dependent of sex.

### Considerations

The GWAS conducted in UKB was not filtered on European ancestry, meaning that the analyses included ∼5% non-Europeans. As the allele frequencies could vary in the different populations, this could affect the results. However, we included the first 10 PCs as covariates in the GWAS, which should account for some of the variability from including non-Europeans. All HUNT participants included in this study were of European ancestry. An inherent limitation when performing sex-specific analyses is smaller sample sizes and thus less statistical power. However, there are significant sex differences in CVD prevalence, presentation, and outcome, indicating sex-specific pathophysiologic features that requires a sex-based research approach.

In the PRS analyses, there were several null findings. For all-cause mortality and CVD, we are aggregating diseases with large differences in biological mechanisms and pathology. It is likely that RHR variants exert different effects on different diseases through distinct biological pathways, making it hard to detect associations in such heterogenic phenotypes. For CVD, the protective effect on AF and risk increasing effects on hypertension and DCM might dissipate the total association. Furthermore, we found no evidence for an association with stroke, which is interesting as AF and hypertension are important risk factors of stroke.^67,68^ The recent study by van de Vegte *et al*.^14^ suggested an inverse association between genetically predicted RHR and any stroke, ischaemic stroke, and cardio-embolic stroke, but this latter association was attenuated by AF. This might imply that the observed association between genetically predicted RHR and stroke is mediated through AF. We reduced the phenotypical heterogeneity by differentiating between ischaemic and non-ischaemic stroke, but this stratification may be insufficient recognizing the diverse array of potential underlying causes of a stroke.^69^ The lack of association to HF is also noteworthy as RHR has been shown to be associated with higher risk of HF^70^ and in addition, hypertension is the leading cause of HF and HF is found to have a mutual causal relationship with AF.^71^ It may be that there is no genetic link between RHR and HF, but another explanation could be that the heterogeneity of the HF phenotype hampers the identification of an association. Perhaps the link between RHR and HF is only explained by a certain type of HF or is mediated through hypertension. In general, differentiating between subgroups of disease (like HF) would lead to smaller sample sizes and thereby reduce statistical power.

The study population is predominantly of European ancestry, and our findings cannot be generalized to populations of other ethnic backgrounds. We encourage future research to tests the genome-wide PRS in other cohorts to assess the generalizability of our findings.

## Conclusion

We have identified 403, 253, and 167 independent significant SNPs in the total population, women, and men, respectively. The GWAS SNPs were mapped to 575, 355, and 263 genes, and the functional annotation analyses pointed to their enrichment in heart tissue and cardiac conduction pathways, as well as an overrepresentation in disease-pathways, including cardiomyopathy. The sex-specified analyses indicated differences in the genetic contribution to RHR and revealed RHR loci specific to one sex. The PRS analyses demonstrated that a high genetically predicted RHR was associated with an increased risk of hypertension and DCM and a decreased risk of AF. Even though the underlying mechanisms linking RHR and these diseases are not fully elucidated, the RHR PRS developed in this study can to some extent predict the disease risk. Our genome-wide meta-analysis and PRS have shed light on the pleiotropic effects of the RHR variants, and the sex-specific results could contribute to a more refined understanding of RHR as a risk factor for the different diseases.

## Supplementary Material

zwae092_Supplementary_Data

## Data Availability

The summary statistics from the genome-wide meta-analysis and the PRS weight file will be made available in an online repository at publication.

## References

[1] Townsend N , Kazakiewicz D, Lucy Wright F, Timmis A, Huculeci R, Torbica A, et al Epidemiology of cardiovascular disease in Europe. Nat Rev Cardiol 2022;19:133–143. 34497402 10.1038/s41569-021-00607-3

[2] Cooney MT , Vartiainen E, Laatikainen T, Juolevi A, Dudina A, Graham IM et al Elevated resting heart rate is an independent risk factor for cardiovascular disease in healthy men and women. Am Heart J 2010;159:612–619.e3. 20362720 10.1016/j.ahj.2009.12.029

[3] Hu L , Huang X, Zhou W, You C, Liang Q, Zhou D, et al Associations between resting heart rate, hypertension, and stroke: a population-based cross-sectional study. J Clin Hypertens (Greenwich) 2019;21:589–597. 30950555 10.1111/jch.13529 PMC8030446

[4] Fox K , Bousser MG, Amarenco P, Chamorro A, Fisher M, Ford I, et al Heart rate is a prognostic risk factor for myocardial infarction: a post hoc analysis in the PERFORM (Prevention of cerebrovascular and cardiovascular Events of ischemic origin with teRutroban in patients with a history oF ischemic strOke or tRansient ischeMic attack) study population. Int J Cardiol 2013;168:3500–3505. 23706327 10.1016/j.ijcard.2013.04.206

[5] Liu X , Guo N, Zhu W, Zhou Q, Liu M, Chen C, et al Resting heart rate and the risk of atrial fibrillation. Int Heart J 2019;60:805–811. 31204373 10.1536/ihj.18-470

[6] Puig E , Clará A, Pérez S, Degano IR, Subirana I, García-García C, et al Resting heart rate, cardiovascular events, and all-cause mortality: the REGICOR study. Eur J Prev Cardiol 2022;29:e200–e202. 34477860 10.1093/eurjpc/zwab148

[7] Aune D , Sen A, Ó'hartaigh B, Janszky I, Romundstad PR, Tonstad S, et al Resting heart rate and the risk of cardiovascular disease, total cancer, and all-cause mortality—a systematic review and dose-response meta-analysis of prospective studies. Nutr Metab Cardiovasc Dis 2017;27:504–517. 28552551 10.1016/j.numecd.2017.04.004

[8] Olshansky B , Ricci F, Fedorowski A. Importance of resting heart rate. Trends Cardiovasc Med 2022;33:502–515. 35623552 10.1016/j.tcm.2022.05.006

[9] Jensen MT . Resting heart rate and relation to disease and longevity: past, present and future. Scand J Clin Lab Invest 2019;79:108–116. 30761923 10.1080/00365513.2019.1566567

[10] Singh JP , Larson MG, O’Donnell CJ, Tsuji H, Evans JC, Levy D. Heritability of heart rate variability. Circulation 1999;99:2251–2254. 10226089 10.1161/01.cir.99.17.2251

[11] Tegegne BS , Man T, Roon AMV, Asefa NG, Riese H, Nolte I, et al Heritability and the genetic correlation of heart rate variability and blood pressure in >29 000 families. Hypertension 2020;76:1256–1262. 10.1161/HYPERTENSIONAHA.120.15227 PMC7480943 32829661

[12] Munroe PB , Tinker A. Heritability of resting heart rate and association with mortality in middle-aged and elderly twins. Heart 2018;104:6–7. 28659304 10.1136/heartjnl-2017-311657

[13] Guo Y , Chung W, Zhu Z, Shan Z, Li J, Liu S, et al Genome-wide assessment for resting heart rate and shared genetics with cardiometabolic traits and type 2 diabetes. J Am Coll Cardiol 2019;74:2162–2174. 31648709 10.1016/j.jacc.2019.08.1055

[14] van de Vegte YJ , Eppinga RN, van der Ende MY, Hagemeijer YP, Mahendran Y, Salfati E, et al Genetic insights into resting heart rate and its role in cardiovascular disease. Nat Commun 2023;14:4646. 37532724 10.1038/s41467-023-39521-2 PMC10397318

[15] Xie T , Wang B, Nolte IM, van der Most PJ, Oldehinkel AJ, Hartman CA, et al Genetic risk scores for complex disease traits in youth. Circ Genom Precis Med 2020;13:e002775. 32527150 10.1161/CIRCGEN.119.002775 PMC7439939

[16] Vilhjálmsson BJ , Yang J, Finucane HK, Gusev A, Lindström S, Ripke S, et al Modeling linkage disequilibrium increases accuracy of polygenic risk scores. Am J Hum Genet 2015;97:576–592. 26430803 10.1016/j.ajhg.2015.09.001 PMC4596916

[17] Krokstad S , Langhammer A, Hveem K, Holmen T, Midthjell K, Stene T, et al Cohort profile: the HUNT study, Norway. Int J Epidemiol 2013;42:968–977. 22879362 10.1093/ije/dys095

[18] Brumpton BM , Graham S, Surakka I, Skogholt AH, Løset M, Fritsche LG, et al The HUNT study: a population-based cohort for genetic research. Cell Genomics 2022;2:100193. 36777998 10.1016/j.xgen.2022.100193 PMC9903730

[19] Ferreira MA , Vonk JM, Baurecht H, Marenholz I, Tian C, Hoffman JD, et al Shared genetic origin of asthma, hay fever and eczema elucidates allergic disease biology. Nat Genet 2017;49:1752–1757. 29083406 10.1038/ng.3985 PMC5989923

[20] Sudlow C , Gallacher J, Allen N, Beral V, Burton P, Danesh J, et al UK Biobank: an open access resource for identifying the causes of a wide range of complex diseases of middle and old age. PLoS Med 2015;12:e1001779. 25826379 10.1371/journal.pmed.1001779 PMC4380465

[21] Loh P-R , Tucker G, Bulik-Sullivan BK, Vilhjálmsson BJ, Finucane HK, Salem RM, et al Efficient Bayesian mixed-model analysis increases association power in large cohorts. Nat Genet 2015;47:284–290. 25642633 10.1038/ng.3190 PMC4342297

[22] Willer CJ , Li Y, Abecasis GR. METAL: fast and efficient meta-analysis of genomewide association scans. Bioinformatics 2010;26:2190–2191. 20616382 10.1093/bioinformatics/btq340 PMC2922887

[23] Watanabe K , Taskesen E, van Bochoven A, Posthuma D. Functional mapping and annotation of genetic associations with FUMA. Nat Commun 2017;8:1826. 29184056 10.1038/s41467-017-01261-5 PMC5705698

[24] Ge T , Chen C-Y, Ni Y, Feng Y-CA, Smoller JW. Polygenic prediction via Bayesian regression and continuous shrinkage priors. Nat Commun 2019;10:1776. 30992449 10.1038/s41467-019-09718-5 PMC6467998

[25] Niiranen TJ , McCabe EL, Larson MG, Henglin M, Lakdawala NK, Vasan RS, et al Heritability and risks associated with early onset hypertension: multigenerational, prospective analysis in the Framingham Heart Study. Bmj 2017;357:j1949. 28500036 10.1136/bmj.j1949 PMC5430541

[26] Niiranen TJ , Larson MG, McCabe EL, Xanthakis V, Vasan RS, Cheng S. Prognosis of prehypertension without progression to hypertension. Circulation 2017;136:1262–1264. 28947482 10.1161/CIRCULATIONAHA.117.029317 PMC5658013

[27] Buck C , Baker P, Bass M, Donner A. The prognosis of hypertension according to age at onset. Hypertension 1987;9:204–208. 3818017 10.1161/01.hyp.9.2.204

[28] Palatini P , Dorigatti F, Zaetta V, Mormino P, Mazzer A, Bortolazzi A, et al Heart rate as a predictor of development of sustained hypertension in subjects screened for stage 1 hypertension: the HARVEST Study. J Hypertens 2006;24:1873–1880. 16915038 10.1097/01.hjh.0000242413.96277.5b

[29] Dalal J , Dasbiswas A, Sathyamurthy I, Maddury SR, Kerkar P, Bansal S, et al Heart rate in hypertension: review and expert opinion. Int J Hypertens 2019;2019:2087064. 30915238 10.1155/2019/2087064 PMC6399539

[30] Eppinga RN , Hagemeijer Y, Burgess S, Hinds DA, Stefansson K, Gudbjartsson DF, et al Identification of genomic loci associated with resting heart rate and shared genetic predictors with all-cause mortality. Nat Genet 2016;48:1557–1563. 27798624 10.1038/ng.3708

[31] Grassi G , Vailati S, Bertinieri G, Seravalle G, Stella ML, Dell'Oro R, et al Heart rate as marker of sympathetic activity. J Hypertens 1998;16:1635–1639. 9856364 10.1097/00004872-199816110-00010

[32] Grassi G . Sympathetic overdrive and cardiovascular risk in the metabolic syndrome. Hypertens Res 2006;29:839–847. 17345783 10.1291/hypres.29.839

[33] Whelton SP , Blankstein R, Al-Mallah MH, Lima JAC, Bluemke DA, Hundley WG, et al Association of resting heart rate with carotid and aortic arterial stiffness. Hypertension 2013;62:477–484. 23836802 10.1161/HYPERTENSIONAHA.113.01605 PMC3838105

[34] Siland JE , Geelhoed B, Roselli C, Wang B, Lin HJ, Weiss S, et al Resting heart rate and incident atrial fibrillation: a stratified Mendelian randomization in the AFGen consortium. PLOS ONE 2022;17:e0268768. 35594314 10.1371/journal.pone.0268768 PMC9122202

[35] Mensah-Kane J , Schmidt AF, Hingorani AD, Finan C, Chen Y, van Duijvenboden S, et al No clinically relevant effect of heart rate increase and heart rate recovery during exercise on cardiovascular disease: a Mendelian randomization analysis. Front Genet 2021;12:569323. 33679875 10.3389/fgene.2021.569323 PMC7931909

[36] Larsson SC , Drca N, Mason AM, Burgess S. Resting heart rate and cardiovascular disease. Circ Genom Precis Med 2019;12:e002459. 30919689 10.1161/CIRCGEN.119.002459 PMC7612931

[37] Klevjer M , Rasheed H, Romundstad PR, Madssen E, Brumpton BM, Bye A et al Insight into the relationship between resting heart rate and atrial fibrillation: a Mendelian randomization study. EP Europace 2023;25:euad292. 37738632 10.1093/europace/euad292 PMC10551233

[38] Sigurdsson MI , Waldron NH, Bortsov AV, Smith SB, Maixner W. Genomics of cardiovascular measures of autonomic tone. J Cardiovasc Pharmacol 2018;71:180–191. 29300220 10.1097/FJC.0000000000000559 PMC5839974

[39] Guasch E , Benito B, Qi X, Cifelli C, Naud P, Shi Y, et al Atrial fibrillation promotion by endurance exercise: demonstration and mechanistic exploration in an animal model. J Am Coll Cardiol 2013;62:68–77. 23583240 10.1016/j.jacc.2013.01.091

[40] Myrstad M , Løchen ML, Graff-Iversen S, Gulsvik AK, Thelle DS, Stigum H, et al Increased risk of atrial fibrillation among elderly Norwegian men with a history of long-term endurance sport practice. Scand J Med Sci Sports 2014;24:e238–e244. 24256074 10.1111/sms.12150 PMC4282367

[41] Johansen KR , Ranhoff AH, Sørensen E, Nes BM, Heitmann KA, Apelland T, et al Risk of atrial fibrillation and stroke among older men exposed to prolonged endurance sport practice: a 10-year follow-up. The Birkebeiner Ageing Study and the Tromsø Study. Open Heart 2022;9:e002154. 36396296 10.1136/openhrt-2022-002154 PMC9677011

[42] Morseth B , Løchen ML, Ariansen I, Myrstad M, Thelle DS. The ambiguity of physical activity, exercise and atrial fibrillation. Eur J Prev Cardiol 2018;25:624–636. 29411631 10.1177/2047487318754930

[43] Karjalainen J , Kujala UM, Kaprio J, Sarna S, Viitasalo M. Lone atrial fibrillation in vigorously exercising middle aged men: case–control study. Bmj 1998;316:1784–1785. 9624065 10.1136/bmj.316.7147.1784 PMC28577

[44] Elliott AD , Linz D, Verdicchio CV, Sanders P. Exercise and atrial fibrillation: prevention or causation? Heart Lung Circ 2018;27:1078–1085. 29891251 10.1016/j.hlc.2018.04.296

[45] McKenna WJ , Maron BJ, Thiene G. Classification, epidemiology, and global burden of cardiomyopathies. Circ Res 2017;121:722–730. 28912179 10.1161/CIRCRESAHA.117.309711

[46] Gupta S , Figueredo VM. Tachycardia mediated cardiomyopathy: pathophysiology, mechanisms, clinical features and management. Int J Cardiol 2014;172:40–46. 24447747 10.1016/j.ijcard.2013.12.180

[47] Jordan E , Peterson L, Ai T, Asatryan B, Bronicki L, Brown E, et al Evidence-based assessment of genes in dilated cardiomyopathy. Circulation 2021;144:7–19. 33947203 10.1161/CIRCULATIONAHA.120.053033 PMC8247549

[48] Ciarambino T , Menna G, Sansone G, Giordano M. Cardiomyopathies: an overview. Int J Mol Sci 2021;22:7722. 34299342 10.3390/ijms22147722 PMC8303989

[49] Pierre SR S , Peirlinck M, Kuhl E. Sex matters: a comprehensive comparison of female and male hearts. Front Physiol 2022;13:831179. 35392369 10.3389/fphys.2022.831179 PMC8980481

[50] James AF , Choisy SCM, Hancox JC. Recent advances in understanding sex differences in cardiac repolarization. Prog Biophys Mol Biol 2007;94:265–319. 15979693 10.1016/j.pbiomolbio.2005.05.010

[51] Peirlinck M , Sahli Costabal F, Kuhl E. Sex differences in drug-induced arrhythmogenesis. Front Physiol 2021;12:708435. 34489728 10.3389/fphys.2021.708435 PMC8417068

[52] Willemars MMA , Nabben M, Verdonschot JAJ, Hoes MF. Evaluation of the interaction of sex hormones and cardiovascular function and health. Curr Heart Fail Rep 2022;19:200–212. 35624387 10.1007/s11897-022-00555-0 PMC9329157

[53] Bernabeu E , Canela-Xandri O, Rawlik K, Talenti A, Prendergast J, Tenesa A. Sex differences in genetic architecture in the UK Biobank. Nat Genet 2021;53:1283–1289. 34493869 10.1038/s41588-021-00912-0

[54] Bruneau BG . The developmental genetics of congenital heart disease. Nature 2008;451:943–948. 18288184 10.1038/nature06801

[55] González-Castro TB , Tovilla-Zárate CA, López-Narvaez ML, Juárez-Rojop IE, Calderón-Colmenero J, Sandoval JP, et al Association between congenital heart disease and NKX2.5 gene polymorphisms: systematic review and meta-analysis. Biomark Med 2020;14:1747–1757. 33346701 10.2217/bmm-2020-0190

[56] Dargis N , Lamontagne M, Gaudreault N, Sbarra L, Henry C, Pibarot P, et al Identification of gender-specific genetic variants in patients with bicuspid aortic valve. Am J Cardiol 2016;117:420–426. 26708639 10.1016/j.amjcard.2015.10.058

[57] Harvey RD . Muscarinic receptor agonists and antagonists: effects on cardiovascular function. In: Fryer AD Christopoulos A and Nathanson NM (eds.), Muscarinic receptors. Berlin, Heidelberg: Springer Berlin Heidelberg; 2012. p299–316. 10.1007/978-3-642-23274-9_13 22222704

[58] Fu LX , Magnusson Y, Bergh CH, Liljeqvist JA, Waagstein F, Hjalmarson A, et al Localization of a functional autoimmune epitope on the muscarinic acetylcholine receptor-2 in patients with idiopathic dilated cardiomyopathy. J Clin Invest 1993;91:1964–1968. 7683693 10.1172/JCI116416 PMC288192

[59] Nussinovitch U , Shoenfeld Y. The diagnostic and clinical significance of anti-muscarinic receptor autoantibodies. Clin Rev Allergy Immunol 2012;42:298–308. 21207192 10.1007/s12016-010-8235-x

[60] Ma G , Wu X, Zeng L, Jin J, Liu X, Zhang J, et al Association of autoantibodies against M2-muscarinic acetylcholine receptor with atrial fibrosis in atrial fibrillation patients. Cardiol Res Pract 2019;2019:8271871. 30863630 10.1155/2019/8271871 PMC6378765

[61] Russell FA , King R, Smillie SJ, Kodji X, Brain SD. Calcitonin gene-related peptide: physiology and pathophysiology. Physiol Rev 2014;94:1099–1142. 25287861 10.1152/physrev.00034.2013 PMC4187032

[62] Valentini A , Petraglia F, De Vita D, Margutti A, degli Uberti EC, Genazzani AR. Changes of plasma calcitonin gene-related peptide levels in postmenopausal women. Am J Obstet Gynecol 1996;175:638–642. 8828427 10.1053/ob.1996.v175.a74287

[63] Peroni RN , Abramoff T, Neuman I, Podestá EJ, Adler-Graschinsky E. Phytoestrogens enhance the vascular actions of the endocannabinoid anandamide in mesenteric beds of female rats. Int J Hypertens 2012;2012:647856. 22319644 10.1155/2012/647856 PMC3272812

[64] Shao A , Shi J, Liang Z, Pan L, Zhu W, Liu S, et al Meta-analysis of the association between Apolipoprotein E polymorphism and risks of myocardial infarction. BMC Cardiovasc Disord 2022;22:126. 35331149 10.1186/s12872-022-02566-0 PMC8952226

[65] Semaev S , Shakhtshneider E, Shcherbakova L, Orlov P, Ivanoshchuk D, Malyutina S, et al Association of common variants of APOE, CETP, and the 9p21.3 chromosomal region with the risk of myocardial infarction: a prospective study. Int J Mol Sci 2023;24:10908. 37446094 10.3390/ijms241310908 PMC10342168

[66] Altmann A , Tian L, Henderson VW, Greicius MD. Sex modifies the APOE-related risk of developing Alzheimer disease. Ann Neurol 2014;75:563–573. 24623176 10.1002/ana.24135 PMC4117990

[67] O'Donnell MJ , Xavier D, Liu L, Zhang H, Chin SL, Rao-Melacini P, et al Risk factors for ischaemic and intracerebral haemorrhagic stroke in 22 countries (the INTERSTROKE study): a case–control study. Lancet 2010;376:112–123. 20561675 10.1016/S0140-6736(10)60834-3

[68] Wolf PA , Abbott RD, Kannel WB. Atrial fibrillation as an independent risk factor for stroke: the Framingham Study. Stroke 1991;22:983–988. 1866765 10.1161/01.str.22.8.983

[69] Feske SK . Ischemic stroke. Am J Med 2021;134:1457–1464. 34454905 10.1016/j.amjmed.2021.07.027

[70] Tadic M , Cuspidi C, Grassi G. Heart rate as a predictor of cardiovascular risk. Eur J Clin Invest 2018;48:e12892. 10.1111/eci.12892 29355923

[71] Zhang Z , Li L, Hu Z, Zhou L, Zhang Z, Xiong Y, et al Causal effects between atrial fibrillation and heart failure: evidence from a bidirectional Mendelian randomization study. BMC Med Genomics 2023;16:187. 37580781 10.1186/s12920-023-01606-8 PMC10424396

