# Supplementary material for: Sex-specific and polygenic effects underlying resting heart rate and associated risk of cardiovascular disease
Source: Eur J Prev Cardiol. 2024 Mar 4;31(13):1585–94. doi: 10.1093/eurjpc/zwae092 (PMC11412739; doi:10.1093/eurjpc/zwae092)
Supplement: zwae092_Supplementary_Data [file zwae092_supplementary_data.zip › Supplementary material.docx]

# Supplementary materials

Sex-specific and polygenic effects underlying resting heart rate and associated risk of cardiovascular disease.

Ada N Nordeidet^1 *^, Marie Klevjer^1,2, *^, Karsten Øvretveit^3^, Erik Madssen^2^, Ulrik Wisløff^1,4^, Ben M Brumpton^3^, Anja Bye^1,2^

1) Cardiac Exercise Research Group (CERG), Department of Circulation and Medical Imaging, Faculty of Medicine and Health Sciences, Norwegian University of Science and Technology (NTNU), Trondheim, Norway

2) Department of Cardiology, St. Olavs Hospital, Trondheim University Hospital, Trondheim, Norway

3) K.G. Jebsen Center for Genetic Epidemiology, Department of Public Health and Nursing, Faculty of Medicine and Health Sciences, Norwegian University of Science and Technology (NTNU), Trondheim, Norway

4) Centre for Research on Exercise, Physical Activity and Health, School of Human Movement and Nutrition Sciences, University of Queensland, St. Lucia, Brisbane, Queensland, Australia.

* These two authors contributed equally

Corresponding author: Ada N Nordeidet

e-mail address: [ada.n.nordeidet@ntnu.no](mailto:ada.n.nordeidet@ntnu.no)

**Supplementary Table 1:** The 10th revision of the international classification of diseases used for classifying disease outcomes.

| Outcome | ICD-10 code(s) |
| --- | --- |
| Hypertension | I10 |
| Early-onset hypertension | I10 < 55 years old |
| Late-onset hypertension | I10 ≥ 55 years old |
| Atrial fibrillation | I48 |
| Dilated cardiomyopathy | I42.0 |
| Hypertrophic cardiomyopathy | I42.1, I42.2 |
| Heart Failure | I50.0, I50.1, I50.9 |
| Stroke | I60.0, I60.1, I60.2, I60.3, I60.4, I60.5, I60.6, I60.7, I60.8, I60.9, I61.0, I61.1, I61.2, I61.3, I61.4, I61.5, I61.6, I61.8, I61.9, I62.0, I62.1, I62.9, I63.0, I63.1, I63.2, I63.3, I63.4, I63.5, I63.6, I63.8, I63.9, I64 |
| Non-ischemic stroke | I60.0, I60.1, I60.2, I60.3, I60.4, I60.5, I60.6, I60.7, I60.8, I60.9, I61.0, I61.1, I61.2, I61.3, I61.4, I61.5, I61.6, I61.8, I61.9, I62.0, I62.1, I62.9 |
| Ischemic stroke | I63.0, I63.1, I63.2, I63.3, I63.4, I63.5, I63.6, I63.8, I63.9 |
| Myocardial infarction | I21.0, I21.01, I21.02, I21.03, I21.0a, I21.0b, I21.1, I21.11, I21.13, I21.1a, I21.2, I21.21, I21.3, I21.31, I21.32, I21.3b, I21.4, I21.41, I21.42, I21.4a, I21.4b, I21.9, I21.91, I21.92, I21.9a |
| Cardiovascular disease | I11.0, I11.9, I13.0, I13.1, I13.2, I13.9, I20.0, I20.1, I20.8, I20.9, I21.0, I21.01, I21.02, I21.03, I21.0a, I21.0b, I21.1, I21.11, I21.13, I21.1a, I21.2, I21.21, I21.3, I21.31, I21.32, I21.3b, I21.4, I21.41, I21.42, I21.4a, I21.4b, I21.9, I21.91, I21.92, I21.9a, I24.8, I24.9, I25.0, I25.1, I25.2, I25.3, I25.4, I25.5, I25.6, I25.8, I25.9,I26.0, I26.9, I27.0, I27.2, I27.8, I27.9, I34.0, I34.1, I34.2, I34.8, I34.9, I35.0, I35.1, I35.2, I35.8, I35.9, I36.0, I36.1, I36.2, I36.8, I36.9, I37.0, I37.1, I37.8, I37.9, I42.0, I42.1, I42.2, I42.9, I44.0, I44.1, I44.2, I44.3, I44.4, I44.5, I44.6, I44.7, I45.0, I45.1, I45.2, I45.3, I45.4, I45.5, I45.6, I45.8, I45.9, I46.0, I46.1, I46.9, I47.0, I47.1, I47.2, I47.9, I48, I48.0, I48.1, I48.2, I48.3, I48.4, I48.9, I49.0, I49.1, I49.2, I49.3, I49.4, I49.5, I49.8, I49.9, I50.0, I50.1, I50.9, I51.0, I51.1, I51.2, I51.3, I51.4, I51.5, I51.6, I51.7, I51.8, I51.9, I60.0, I60.1, I60.2, I60.3, I60.4, I60.5, I60.6, I60.7, I60.8, I60.9, I61.0, I61.1, I61.2, I61.3, I61.4, I61.5, I61.6, I61.8, I61.9, I62.0, I62.1, I62.9, I63.0, I63.1, I63.2, I63.3, I63.4, I63.5, I63.6, I63.8 I63.9, I64, I65.0, I65.1, I65.2, I65.3, I65.8, I65.9, I66.0, I66.1, I66.2, I66.3, I66.4, I66.8, I66.9, I67.0, I67.1, I67.2, I67.3, I67.4, I67.5, I67.6, I67.7, I67.8, I67.9, I68.0, I70.0, I70.00, I70.01, I70.1, I70.10, I70.2, I70.20, I70.21, I70.8, I70.80, I70.81, I70.9, I70.90, I70.91, I71.0, I71.1, I71.2, I71.3, I71.4, I71.5, I71.6, I71.8, I71.9, I72.0, I72.1, I72.2, I72.3, I72.4, I72.5, I72.6, I72.8, I72.9, I73.9, I74.0, I74.1, I74.2, I74.3, I74.4, I74.5, I74.8, I74.9, I82.2, I82.3, I82.8, I82.9 |

**Supplementary Table 2:** Number of controls and cases for all HUNT outcomes.

| **Condition** | **Controls total population** | **Cases total population** | **Cases in women** | **Cases in men** |
| --- | --- | --- | --- | --- |
| Hypertension | 69938 | 16749 | 8774 | 7075 |
| Early-onset hypertension | 28818 | 1951 | 954 | 997 |
| Late-onset hypertension | 41120 | 14798 | 7820 | 6978 |
| Cardiovascular disease | 61384 | 25297 | 12181 | 13116 |
| Myocardial infarction | 81821 | 4866 | 1833 | 3033 |
| Stroke | 81269 | 5418 | 2627 | 2791 |
| Ischemic stroke | 82269 | 4418 | 2132 | 2286 |
| Non-ischemic stroke | 85520 | 1167 | 560 | 607 |
| Atrial fibrillation | 81654 | 5032 | 2143 | 2889 |
| Dilated cardiomyopathy | 86293 | 394 | 118 | 279 |
| Hypertrophic cardiomyopathy | 86305 | 382 | 187 | 195 |
| Heart failure | 81275 | 5412 | 2600 | 2812 |


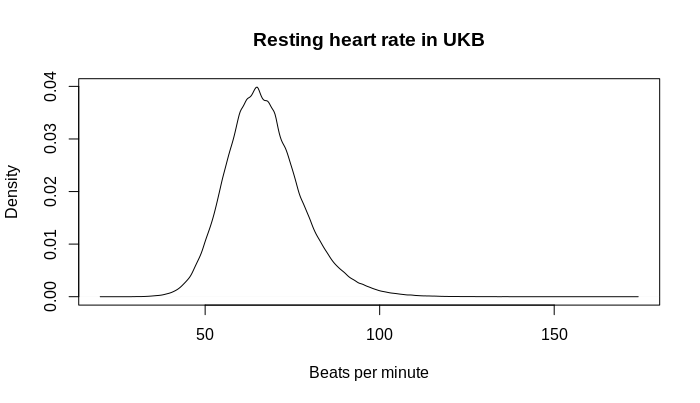

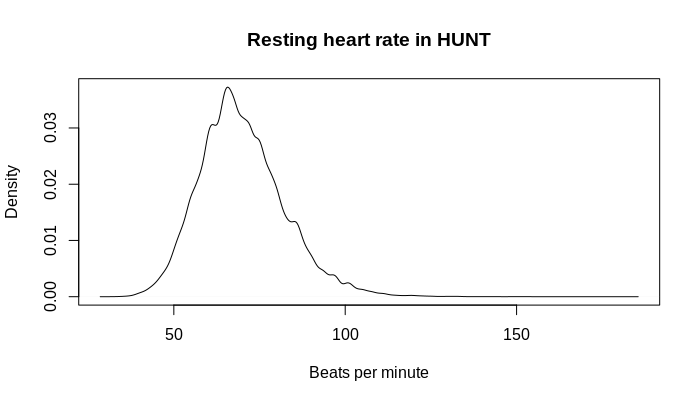


**Supplementary Figure 1:** The distribution of resting heart rate in the UK Biobank to the left and The Trøndelag Health Study to the right. The resting heart rate in beats per minute is on the x-axis, and the density is on the y-axis.

**Supplementary Table 3:** RHR loci only found in men.

| Risk loci number | Position (start-end) | Chr | Top lead SNP | p-value top lead SNP | Effect top lead SNP | Mapped genes |
| --- | --- | --- | --- | --- | --- | --- |
| 3 | 66114119-67437157 | 1 | 1:66991346_T/C (rs10789207) | 1.504e-17 | -0.3807 | LEPR, PDE4B, **SGIP1,** AL139147.1, **TCTEX1D1,** INSL5, WDR78, MIER1 |
| 8 | 159958894-159983331 | 2 | 2:159969067_CT/C | 3.09e-09 | -0.2995 | TANC1 |
| 10 | 187821910-188421264 | 2 | 2:188190475_G/A | 2.324e-14 | -0.3506 | CALCRL, TFPI |
| 22 | 134123317-134226147 | 6 | 6:134179681_T/TGCAGTCC | 1.337e-10 | 0.2817 | TCF21 |
| 23 | 35095787-35306718 | 7 | 7:35240475_T/C | 2.318e-09 | -0.2428 | TBX20 |
| 28 | 35908313-35948588 | 9 | 9:35908313_A/C | 3.014e-08 | -0.2214 | HRCT1, OR2S2 |
| 34 | 62310909-62418921 | 11 | 11:62364678_C/CA | 3.345e-08 | 0.0417 | AHNAK, EEF1G, MIR3654, TUT1, MTA2, EML3, ROM1, B3GAT3, GANAB, INTS5, RP11-831H9.11 |
| 37 | 20383093-20474260 | 12 | 12:20474123_G/A | 1.16e-12 | 0.3282 |  |
| 39 | 27939355-27955296 | 12 | 12:27955296_C/T | 9.728e-10 | 0.2408 | KLHL42 |
| 43 | 82101514-82279404 | 12 | 12:82203236_ACTTTACTTTTAGAATGT/A | 1.379e-10 | 0.3089 | PPFIA2 |
| 45 | 116238229-116247694 | 12 | 12:116238962_TTTTTTG/T | 5.368e-12 | 0.2802 |  |
| 51 | 83584643-83670073 | 15 | 15:83663887_TG/T | 1.13e-08 | 0.2266 | HOMER2, FAM103A1, C15orf40 |
| 54 | 19982353-20054371 | 16 | 16:20045303_G/GTT | 3.666e-08 | -0.2659 | GPR139 |
| 57 | 20063670-20129524 | 18 | 18:20070864_A/AT | 2.751e-14 | 0.6943 |  |
| 58 | 26259012-26454930 | 18 | 18:26267775_CA/C | 1.207e-10 | -0.2571 |  |
| 60 | 5036564-5088080 | 19 | 19:5056893_GCTGGGGCGGGGAGTC/G | 1.9e-08 | 0.2399 | KDM4B |
| 61 | 36164973-36164973 | 19 | 19:36164973_T/A | 9.946e-09 | -1.7046 | UPK1A |
| 62 | 40703746-40866722 | 19 | 19:40822873_G/GTA | 1.966e-08 | 0.2274 | MAP3K10, TTC9B, CNTD2, AKT2, C19orf47, PLD3 |
| 63 | 45412079-45426792 | 19 | 19:45413233_G/T | 2.078e-12 | -0.5134 | TOMM40, APOE, APOC1 |
| 64 | 25201575-26176538 | 20 | 20:25422978_G/A | 5.226e-09 | 0.5144 | ENTPD6, AL035252.1, PYGB, ABHD12, GINS1, NINL, NANP, FAM182B |

**Supplementary Table 4:** RHR loci only found in women.

| Risk loci number | Position (start-end) | Chr | Top lead SNP | p-value top lead SNP | Effect top lead SNP | Mapped genes |
| --- | --- | --- | --- | --- | --- | --- |
| 1 | 6278414-6296238 | 1 | 1:6296238_T/G | 9.126e-10 | 0.1997 | RPL22, RNF207, ICMT, HES3 |
| 3 | 39942242-40088043 | 1 | 1:39954548_A/AAT | 3.357e-09 | 0.2473 | MACF1, BMP8A, PABPC4, HEYL |
| 6 | 156434703-156474929 | 1 | 1:156474929_G/GC | 4.307e-10 | 0.2148 | MEF2D |
| 8 | 216731498-216816223 | 1 | 1:216734001_T/A | 1.367e-08 | 0.2339 | ESRRG |
| 10 | 19792759-19882115 | 2 | 2:19870341_C/T | 3.947e-08 | -0.1954 |  |
| 12 | 60002572-60053727 | 2 | 2:60027233_C/CG | 1.095e-10 | 0.2244 |  |
| 13 | 66725062-66725062 | 2 | 2:66725062_T/C | 1.112e-10 | 0.2281 | MEIS1 |
| 15 | 201092279-201244261 | 2 | 2:201170509_CA/C | 2.815e-13 | 0.2527 | SPATS2L |
| 17 | 228126494-228231432 | 2 | 2:228143966_TTTG/T | 2.439e-11 | 0.2351 | COL4A3, MFF, TM4SF20 |
| 22 | 53253182-53542004 | 3 | 3:53519770_C/CT | 3.902e-10 | 0.2271 | TKT, DCP1A, CACNA1D |
| 23 | 69365528-69430007 | 3 | 3:69412154_A/T | 1.194e-10 | 0.2451 | FRMD4B |
| 25 | 141081497-141154542 | 3 | 3:141112859_CTT/C | 1.793e-09 | 0.2022 | ZBTB38 |
| 26 | 156547771-156697097 | 3 | 3:156607089_G/GA | 4.168e-08 | 0.1891 | LEKR1 |
| 27 | 171759410-171833266 | 3 | 3:171759410_C/CT | 1.38e-16 | -0.2796 |  |
| 28 | 172120816-172164990 | 3 | 3:172126618_C/T | 9.475e-09 | 0.1832 | GHSR |
| 31 | 83149641-83293033 | 4 | 4:83254647_T/TA | 4.709e-09 | 0.2395 | HNRNPD |
| 32 | 148970403-149345608 | 4 | 4:148974602_T/C | 1.457e-12 | -0.3045 | ARHGAP10, NR3C2 |
| 33 | 75041362-75123529 | 5 | 5:75063620_C/CAAT | 4.87e-08 | 0.1824 |  |
| 34 | 79025157-79041057 | 5 | 5:79038927_T/C | 4.447e-08 | 0.2497 | CMYA5 |
| 35 | 89236741-89430773 | 5 | 5:89392719_T/TCA | 3.515e-09 | 0.6958 |  |
| 36 | 121866990-121874184 | 5 | 5:121868475_T/TA | 7.592e-10 | 0.222 |  |
| 38 | 142620736-142790449 | 5 | 5:142627256_T/TTGGA | 8.871e-10 | 0.2966 | NR3C1 |
| 39 | 172473495-172677991 | 5 | 5:172653978_C/CT | 3.855e-21 | 0.4509 | CREBRF, BNIP1, NKX2-5 |
| 40 | 7518915-7545081 | 6 | 6:7538279_G/C | 2.413e-08 | -0.3086 | DSP |
| 41 | 22109189-22144059 | 6 | 6:22136160_C/T | 2.532e-09 | 0.1907 |  |
| 42 | 36771587-36896828 | 6 | 6:36817113_A/G | 5.917e-16 | 0.2711 | CPNE5, PPIL1, C6orf89 |
| 47 | 116327430-116541935 | 7 | 7:116432139_A/AT | 2.543e-11 | -0.2221 | MET, CAPZA2 |
| 49 | 130960827-131213850 | 7 | 7:130965408_AT/A | 5.671e-28 | 0.3737 | MKLN1, PODXL |
| 50 | 136560679-136681356 | 7 | 7:136595547_A/AGT | 8.482e-24 | 0.4573 | CHRM2 |
| 51 | 99084471-99282269 | 9 | 9:99165257_C/CT | 4.819e-08 | 0.2396 | SLC35D2, ZNF367, HABP4, CDC14B |
| 54 | 18598390-18720109 | 10 | 10:18646118_C/A | 6.155e-09 | 0.2968 | CACNB2 |
| 55 | 29789738-29824188 | 10 | 10:29821089_C/T | 6.368e-10 | -0.2009 | SVIL |
| 57 | 13998502-14122134 | 11 | 11:14063941_G/GA | 4.247e-13 | -0.2406 |  |
| 63 | 26291149-26348429 | 12 | 12:26345830_CCTA/C | 1.847e-11 | 0.2447 | SSPN |
| 70 | 52454636-52572577 | 14 | 14:52542565_CAT/C | 1.845e-08 | -0.2633 | GNG2, C14orf166, NID2 |
| 73 | 91565706-91617644 | 14 | 14:91584821_AC/A | 6.056e-10 | -0.2489 | C14orf159 |
| 75 | 74247784-74322431 | 15 | 15:74282833_G/GC | 8.089e-09 | 0.2015 | LOXL1, STOML1, PML |
| 78 | 30571910-31155458 | 16 | 16:30619601_CT/C | 2.997e-10 | 0.2489 | AC002310.13, ZNF764, ZNF688, ZNF785, ZNF689, PRR14, FBRS, SRCAP, PHKG2, C16orf93, RNF40, ZNF629, BCL7C, CTF1, FBXL19, ORAI3  SETD1A, BCKDK, KAT8, PRSS8, PRSS36 |
| 79 | 53428284-53537581 | 16 | 16:53440590_CTT/C | 2.455e-10 | -0.2172 | RBL2, AKTIP |
| 82 | 17105615-17194050 | 17 | 17:17126736_T/TCTGC | 2.58e-11 | 0.2213 | MPRIP, PLD6, RP11-45M22.4, FLCN, COPS3 |
| 84 | 77150792-77166143 |  | 18:77156174_G/A | 4.158e-08 | -0.3014 | NFATC1 |
| 85 | 36521069-36623841 | 19 | 19:36535971_C/CTTTA | 7.2e-10 | -0.3329 | CLIP3, THAP8, WDR62, OVOL3, POLR2I, TBCB, CAPNS1 |
| 86 | 39132275-39250337 | 19 | 19:39246976_G/A | 9.873e-13 | 0.2382 | EIF3K, ACTN4, CAPN12 |
| 88 | 61160673-61186053 | 20 | 20:61165192_T/C | 2.639e-11 | 0.2489 | C20orf166 |
| 89 | 28181044-28181399 | 22 | 22:28181044_CCACA/C | 1.809e-10 | -0.2268 | MN1 |

**Supplementary table 5:** Genomic risk loci associated with resting heart rate identified in the total population.

**Supplementary table 6:** Genomic risk loci associated with resting heart rate identified in women.

**Supplementary table 7:** Genomic risk loci associated with resting heart rate identified in men.

**Supplementary table 8:** Independent lead SNPs at R^2^ = 0.2 identified in the total population.

**Supplementary table 9:** Independent lead SNPs at R^2^ = 0.2 identified in women.

**Supplementary table 10:** Independent lead SNPs at R^2^ = 0.2 identified in men.

**Supplementary table 11:** Mapped genes in the total population.

**Supplementary table 12:** Mapped genes in women.

**Supplementary table 13:** Mapped genes in men.
